# Dynamic contrast optical coherence tomography (DyC-OCT) for label-free live cell imaging

**DOI:** 10.1038/s42003-024-05973-5

**Published:** 2024-03-06

**Authors:** Chao Ren, Senyue Hao, Fei Wang, Abigail Matt, Marcello Magri Amaral, Daniel Yang, Leyao Wang, Chao Zhou

**Affiliations:** 1 Department of Biomedical Engineering, Washington University in St Louis, St. Louis, MO USA; 2 Imaging Science Ph.D. Program, Washington University in St Louis, St. Louis, MO USA; 3 Department of Electrical & Systems Engineering, Washington University in St Louis, St. Louis, MO USA; 4 Biomedical Engineering, Universidade Brasil, Sao Paulo, Brazil; 5 Division of Allergy and Immunology, Washington University School of Medicine, St. Louis, MO USA

**Keywords:** Optical imaging, Image processing

## Abstract

Dynamic contrast optical coherence tomography (DyC-OCT), an emerging imaging method, utilizes fluctuation patterns in OCT signals to enhance contrast, thereby enabling non-invasive label-free volumetric live cell imaging. In this mini review, we explain the core concepts behind DyC-OCT image formation and its system configurations, serving as practical guidance for future DyC-OCT users. Subsequently, we explore its applications in delivering high-quality, contrast-enhanced images of cellular morphology, as well as in monitoring changes in cellular activity/viability assay experiments.

## Abstract (web summary)

Dynamic contrast optical coherence tomography (DyC-OCT), an emerging imaging method, utilizes fluctuation patterns in OCT signals to enhance contrast, thereby enabling non-invasive label-free volumetric live cell imaging.

## Introduction

Optical coherence tomography (OCT)^1^, is an optical imaging modality which offers millimeter-depth volumetric optical biopsies of live biological samples within seconds^2,3^. OCT identifies depth-resolved back-scattered light, which arises from the inherent interaction of light with biological tissue, to generate label-free microtomographic images of biological specimens. Despite the prevailing focus of current OCT applications on structural and tissue-level imaging, its micron-level spatial resolution holds the potential for live cell imaging. However, the primary challenge lies in the low contrast resulting from the similarities in optical properties between cellular and subcellular features.

To overcome this problem, researchers have developed various contrast enhancement strategies. Contrast agents, such as microspheres^4^, magnetic microparticles^5^, and gold nanoparticles^6–8^, have been injected into samples to enhance their scattering and make targeted structures of interest stand out. An alternative label-free approach for enhancing cellular features involves the analysis of temporal OCT signals. The signal exhibits fluctuations corresponding to variations in the optical back-scattering properties in each voxel of the sample. These fluctuations reflect movements related to cellular motion, metabolic processes, and other cellular activities. OCT-angiography (OCT-A)^9–11^ and speckle variance OCT (SV-OCT)^12–14^ first applied this label-free contrast analysis to highlight blood vessels and generate microvascular maps. In repeated images of the same tissue, the differences between repeated scans can distinguish areas where blood flows. Recently, this idea has been extended to live cell imaging, using a technique called dynamic contrast OCT (DyC-OCT)^15^. DyC-OCT highlights cell clusters or even individual cells to enhance the contrast of conventional OCT images, revealing the cellular morphology and dynamic information behind them.

This mini-review will cover the principles, implementations, and applications of DyC-OCT. We first provide a brief explanation of the sources of the DyC-OCT signal and detailed data processing procedures. Then, we summarize practical recommendations for data collection and selection of the most suitable system configuration. The scope of our review will extend to the most recent implementations of DyC-OCT, with an emphasis on advancements in morphological visualization and potential activity/viability assays. Furthermore, we will discuss the current challenges of DyC-OCT and propose potential solutions.

## Principles of DyC-OCT

A healthy live cell experiences a variety of activities during its lifecycle, from inner metabolic processes, such as ATP-consuming process^15,16^, to outer cell movements. These ever-changing dynamics alter the detected OCT signal. The movement of scattering particles changes the scattering potential of any one voxel in the OCT volumetric data, causing fluctuations in the OCT signal intensity. By conducting repeated OCT scans, time-varying signal fluctuations are captured and then processed using algorithms to produce color-coded maps depicting DyC-OCT motion patterns. DyC-OCT is distinguished from traditional scattering-based OCT through its emphasis on dynamic fluctuations: the motions of microstructures are accented against the motionless regions in the OCT images, enhancing the image contrast.

### Pattern extraction algorithms employed in DyC-OCT

**Fig. 1:**
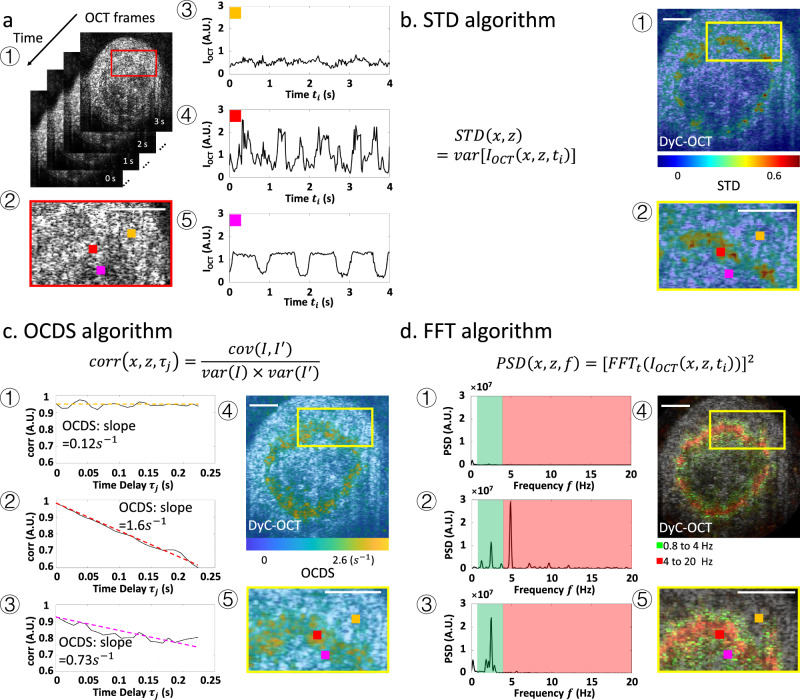
Illustrations of the three DyC-OCT processing algorithms. **a** Repeated OCT frames of airway organoids and OCT time series of three pixels with different dynamics patterns. **a1** Repeated OCT frames of airway organoids. **a2** Zoomed-in view of the red ROI in (**a1**). **a3**–**a5** Normalized time series of OCT signals of the three regions labeled in (**a2**): (**a3**) a stationary region, (**a4**) an intensively moving region, and (**a5**) a mildly moving region. **b** STD based DyC-OCT colormap processing. **b1** STD based color-coded DyC-OCT image of an airway organoid. **b2** Zoomed-in view of the yellow ROI in (**b1**). **c** OCDS based DyC-OCT colormap processing. **c1**–**c3** Autocorrelation by time delay of OCT signals in (**a3**–**a5**); the OCDSs are shown by the slope of the fitted lines of autocorrelations. **c4** OCDS-based color-coded DyC-OCT image of an airway organoid. **c5** Zoomed-in view of the yellow ROI in (**c4**). **d** PSD based DyC-OCT colormap processing. **d1**–**d3** PSDs of OCT signals in (**a3**–**a5**); the green-shaded area represents 0.8–4 Hz, while red-shaded area covers 4–20 Hz. **d4** PSD-based color-coded DyC-OCT image of an airway organoid. **d5** Zoomed-in view of the yellow ROI in (**d4**). *Cov*: covariance; *Var*: variance; ${{FFT}}_{t}$: fast Fourier transform on time dimension. $I={I}_{{OCT}}\left(x,z,{t}_{i}\right),{I}^{{\prime} }={I}_{{OCT}}(x,z,{t}_{i}+{\tau }_{j})\,$. Scale bar: 50 μm.

Various algorithms have been developed to analyze OCT signal fluctuation patterns. Initial approaches, such as the logarithmic intensity variation (LIV)^17–19^ and standard deviation (STD)^15,20–22^ algorithms, utilize the principles in SV-OCT to measure the amplitudes of OCT signal fluctuations. These methods highlight areas of high activity in tissue structures, which exhibit greater fluctuation amplitudes, while reducing the visibility of stationary structures in DyC-OCT colormaps. As an example, Fig. 1a illustrates repeated OCT frames of an airway organoid, a human stem cell-derived 3D assembly that simulates respiratory tissues. The data was obtained using a spectral-domain OCT system, and the organoids were prepared in accordance with the STEMCELL airway organoid culturing protocol^23^. Figure 1.a1 shows the original OCT images, with repeated cross-sectional OCT scans at a fixed location. Each voxel has a temporal series showing its intensity changes. Figure 1.a2 is the zoom-in region marked by the red rectangle in Fig. 1.a1. For the three voxels highlighted in Fig. 1.a2, the OCT signal fluctuation patterns (e.g., amplitude, frequency) are readily distinguishable (Fig. 1.a3–a5). The STD-based DyC-OCT image (Fig. 1.b1) is generated by applying a jet colormap based on the STD value of each pixel. Figure 1.b2 is the same zoom-in region as Fig. 1.a2. Compared to original OCT images, STD-based DyC-OCT colormaps more effectively differentiate structures exhibiting active movements, thereby enhancing image contrast. Although effective, the STD algorithm fails to discern the speed of tissue dynamics. A subsequently development, OCT correlation decay speed (OCDS) analysis^17,18^, overcomes this by measuring the temporal dynamic motion pattern through a temporal decorrelation function (Fig. 1.c). Figure 1.c1–c3 display OCDS measurements from the three pixels indicated in Fig. 1.a2. By measuring the slope of each pixel’s autocorrelation curve (e.g., OCDS value), a direct quantitative assessment of motion dynamics is obtained. Colormaps can be generated using OCDS values (Fig. 1.c4–c5). OCDS-based DyC-OCT analysis can effectively distinguish tissue regions with varying dynamic speeds, offering direct motion dynamics measurements.

Both the STD and OCDS algorithms enhance dynamic contrast in OCT, requiring only around 10 repeated frames^15,17,18^. This facilitates rapid image acquisition and minimizes memory costs and processing time. An additional algorithm, utilizing fast Fourier transform (FFT) to analyze the power spectral density (PSD)^16,24–28^ of each pixel, offers more comprehensive insights into tissue dynamics. Figure 1.d1–d3 demonstrate how PSDs of OCT signals from the pixels shown in Fig. 1.a2 distinctly reveal variations in fluctuation frequencies and intensities. To create a colormap, the integrated intensity within different frequency bands is color-coded^25–27^; for example, signals in the 0.8–4 Hz range are shown in green, while signals in the 4–20 Hz range are shown in red. Furthermore, PSD can highlight dominant frequencies using a continuous colormap^29,30^. These colormaps can be overlaid on structural OCT images to provide a co-registered display of tissue dynamics (Fig. 1.d4–d5). However, while PSD analysis concurrently extracts dynamic signals across frequencies, it requires over 100 repeated OCT scans for accurate PSD measurements, substantially increasing the image acquisition time^16,25,26^. Supplementary Table 1 compares these three DyC-OCT processing algorithms on time complexity, extracted parameters, minimum repeated frames, advantages and drawbacks. Drawing upon established OCT technology, DyC-OCT processing algorithms can identify tissue structures and retrieve both the intensity and speed of their motion patterns. When tailoring DyC-OCT to examine specific biological samples or meet experimental aims, the OCT system setup and acquisition protocol are crucial. We will discuss the system requirements in the next section.

### OCT modalities employed for DyC-OCT

The performance of DyC-OCT is heavily dependent on the choice of OCT hardware. Three types of OCT systems have been applied in DyC-OCT: full-field OCT (FF-OCT), swept-source OCT (SS-OCT), and spectral-domain OCT (SD-OCT). Different OCT modalities present trade-offs in imaging speed, resolution, and depth of penetration, making the selection of an appropriate imaging system and image acquisition parameters critical for optimal performance in specific applications.

FF-OCT^31^ generates OCT images from the *en-face* view using a two-dimensional (2D) camera. It offers a high isotropic resolution (~1 μm) to enable cellular imaging, but its penetration depth in scattering tissue is lower than that of the other two OCT modalities. As a result, FF-OCT is appropriate for samples with limited thickness (~300 μm) demanding superior resolutions, such as the retina organoids and explants^22,24,32^ or sparse cells^28,30^.

SD-OCT and SS-OCT generate cross-sectional OCT images (B-scans) with each acquisition. SD-OCT uses a broadband light source in combination with a spectrometer to achieve a higher axial resolution (~1-2 μm) with an imaging depth of around 1 mm. SD-OCT is well suited for imaging thick tissues with layered structures or depth-resolved features, such as the tongue^31^, trachea^16^, and esophagus^25^. Using a high magnification objective lens, SD-OCT can also achieve ~1 μm lateral resolution with a reduced imaging depth of field^27^. SS-OCT^33^ uses a tunable broadband laser source, a high-speed balanced detector and a data acquisition card to acquire cross-sectional B-scan OCT frames. SS-OCT has a high imaging speed and detection depth that is suitable for imaging thick samples, such as tumor spheroids^17,18^. However, SS-OCT suffers from a low axial resolution of around 10 μm, limiting its ability to image cellular or subcellular structures, such as organelles and nuclei.

Table 1 summarizes the recent literature on DyC-OCT employing different OCT modalities, including information on the OCT system configuration (e.g., system hardware type, resolution, and scan rate), the samples imaged, signal processing algorithms, and the number of repeated frames. The foremost prerequisite for high-quality DyC-OCT analysis is the high spatial resolution: the system must resolve the target microstructure that accounts for added contrast after analysis. Secondly, to meet the Nyquist sampling criteria, the OCT frame or volume rate must be at least twice the frequency of the tissue dynamics being measured. Furthermore, to capture slow tissue dynamic changes, repeated three-dimensional (3D) OCT volumes can be acquired, rather than repeated 2D scans^27^, reducing the total acquisition time for 3D DyC-OCT. For samples exhibiting rapid dynamic changes, ultra-high speed OCT systems are needed to efficiently measure 3D DyC-OCT image stacks. We will discuss about OCT frame or volume rate selection criteria further in Outlook and Conclusions.

**Table 1:** Summary of OCT system parameters, applied samples, and DyC-OCT algorithms used in recent publications

Frame/Volume Rate (Hz)^a^	Resolution (μm)		Samples	Sample FOV (${{{{{\upmu }}}}{{{{{\rm{m}}}}}}}^{3}$) [DyC-OCT Image or Volume Size]^b^		Total Scan Time (s)		Algorithm		${N}_{R}\,$[${S}_{R}$]^c^		Author, Year^ref.^	
	Axial	Lateral											
*FF-OCT: High axial and lateral resolutions; Low penetration depth and fixed FOV*													
138	1	1	mouse brain cortex, rat liver, and kidney	604 × 604 × 42 [-]		20		STD		10 [-]		Apelian et al.^15^	
100	–	0.5	mouse retina, macaque retina	1400 × 1400 [-]		1		FFT		100 [-]		Thouvenin et al.^32^	
100	7.7	1.7	macaque retina	100 × 100 × 100 [-]		–		FFT		100 [-]		Scholler et al.^24^	
–	–	–	esophageal mucosa, duodenal mucosa	1300 × 1300 [-]		12		–		–		Quénéhervé et al.^37^	
100	1.7	0.5	retinal organoids	320 × 320 [1440 × 1440]		–		FFT		512 [-]		Scholler et al.^29^	
300	1 1	5	HeLa cells	–		3.3		FFT		1000 [-]		Park et al.^30^	
–	1 1	5	Nannizzia gypsea, Aspergillus fumigatus, Rhizopus arrhizus	–		–		FFT		–		Maldiney et al.^41^	
100	1.7	0.5	ppRPE and hiRPE	320×320 [-]		–		FFT		512 [-]		Groux et al.^22^	
140	–	–	photosynthetic cells	–		–		FFT		200 [-]		Bey et al.^28^	
*SD-OCT: High axial and lateral resolutions, deep penetration and flexible FOV*													
108	1	1	murine liver, mouse tongue	270 × 285 × 550 [500 × 500 × *N*_*d*_]		695		FFT		100 [500]		Münter et al.^26^	
40	1	2	human esophageal and cervical biopsy	[1000 × *N*_*d*_]		25		FFT		200 [512]		Leung et al.^25^	
24	1.8	1.1	murine trachea wall tissue, murine liver	300 × 300 × 800 [500 × 500 × *N*_d_]		41.7		FFT		100 [500 × 50]		Münter et al.^27^	
20	7.5	7.9	mouse bladder, mouse urothelium	[1000 × *N*_*d*_]		50		STD, OCDS		20 [1000]		Xu et al.^21^	
111	1.5	1.5	mouse trachea	500 × 500 × 800 [500 × 500 × *N*_*d*_]		1200		FFT		150 [500]		Kohlfaerber et al.^16^	
4	3.8	4.9	alveolar organoids	1000 × 1000 × Z [512 × 128 × *N*_*d*_]		60		LIV		32 [512 × 16]		Morishita et al.^43^	
112.5	2.1	3.9	human heart organoids	500 × Z [500 × *N*_*d*_]		–		FFT		[500]		Hao et al.^42^	
*SS-OCT: Deep penetration and flexible FOV; Low axial resolution*													
4	14	19	tumor spheroid	–		52.4		LIV, OCDS		65 [512 × 16]		El-Sadek et al.^17^	
4	14	18.1	tumor spheroid	1000 × 1000 × Z [512 × 128 × *N*_*d*_]		52.4		LIV, OCDS		32 [512 × 16]		El-Sadek et al.^18^	

*FFT* fast Fourier transform, *Amp* amplitude, *OCDS* OCT correlation decay speed, *FOV* field of view, *Z* imaged tissue depth, *N*_*d*_ number of pixels in imaging depth. These two terms are not specified in literatures.

^a^For FF-OCT, the acquired frames are fixed in size. For SD-OCT and SS-OCT, the frame/volume rates are specified with the size of repeated frames/volumes.

^b^For DyC-OCT 3D volumes, FOV is shown in the form of (*W* × *H* × *D*). For DyC-OCT 2D images, FOV is shown in the form of (*W* × *H*). *W* width, *H* height and *D* depth.

^c^*N*_*R*_ Number of repeated OCT frames or volumes acquired for DyC-OCT processing. *S*_*R*_: Data size of each repeated OCT frame or volume. For repeated volumes, the size is shown in the form of *N*_*A*_ × *N*_*B*_. For repeated frames the size is *N*_*A*_. *N*_*A*_ number of A-scans, and *N*_*B*_ number of B-scans.

## Applications of DyC-OCT

DyC-OCT applications can be divided into two types. The first utilizes dynamic information to highlight regions of high temporal variations, producing a morphological image with enhanced contrast. The second type interprets dynamics as an indicator of cell viability, with less concern for structures.

### DyC-OCT to visualize cell and tissue morphology

Several approaches are available for visualizing cell and tissue morphology. Histology^34^, extensively used for disease diagnosis, examines stained and sectioned samples under a microscope in two dimensions. However, it involves tedious and invasive sample preparation methods, which induce inevitable distortion and deteriorate cells from their normal conditions. Fluorescence microscopy^35^ can visualize specific cell structures and physiology with the aid of exogenous or endogenous fluorophores. 2D wide-field epifluorescence^36^ imaging captures features readily, but has limited contrast and spatial resolution, while 3D fluorescence imaging modalities, such as confocal or two-photon microscopy, support depth scanning. However, the slow imaging speed (e.g., 5-10 frames per second) and restricted imaging depth (e.g., a few hundred microns) hinder imaging of large samples. As an alternative, DyC-OCT can achieve non-invasive, cell/tissue-specific, high-speed, and depth-resolvable dynamic live tissue imaging without the need for exogenous contrast agents.

**Fig. 2:**
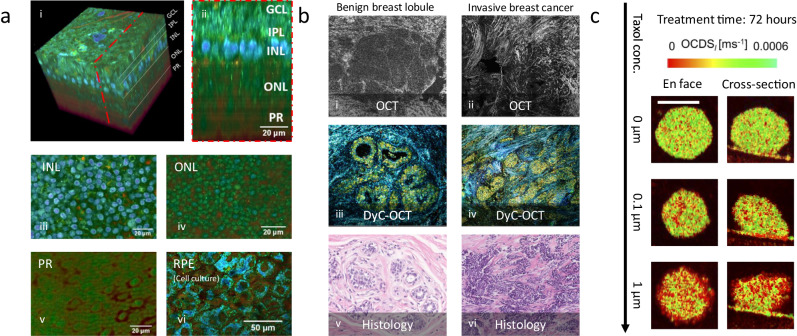
Illustrations of DyC-OCT representative applications. **a** Morphology of retinal cells in different retinal layers^23^. (i) 3D volumetric DyC-OCT cellular morphology of a retinal explant. (ii) cross-sectional DyC-OCT morphology, showing retinal layers. (iii, iv, v, and vi) en face DyC-OCT cellular morphology of the inner nuclear layer (INL), outer nuclear layer (ONL), photoreceptor (PR) layer, and retinal pigment epithelium (RPE) layer. **b** Comparison of a benign breast lobule (i, OCT; iii, DyC-OCT; v, histology) and invasive breast cancer (ii, OCT; iv, DyC-OCT; vi, histology)^40^. **c** Necrosis of tumor spheroids revealed by DyC-OCT with increasing concentrations of Taxol (Paclitaxel)^18^, scale bar: 200 μm. (a), (b), and (c) are reproduced from Scholler et al.^23^, Yang et al.^40^, and El-Sadek et al.^18^ with permissions from publishers.

DyC-OCT supports the identification of high dynamic structures with morphologies resembling cells and subcellular structures (nuclei and organelles) in various organs, such as mouse brain^15^, murine liver^15,26,27^, human esophageal tissues^25^, human gut explants^37^, as well as airway tissues^16^. DyC-OCT demonstrates a remarkable capability to reveal dynamic structures with distinguishable morphologies in different layers of retinal explants^22,24,32^. This matches the fact that retinal explants have different cell types by layers (Fig. 2a), which has been validated by histology^38^ and fluorescence images^39^. DyC-OCT also has impressive potential for cancer diagnoses, since it can detect dynamics in cancerous tissues, such as murine cancerous intestine^15^ and breast cancer^40^. Figure 2b compares OCT, DyC-OCT, and histology images of benign breast lobules and invasive breast tissue, where DyC-OCT dramatically enhances the contrast of OCT, and reports a high diagnostic accuracy^40^. DyC-OCT has also demonstrated the potential of fungal infection diagnosis to visualize 3D structures of Nannizzia gypsea, Aspergillus fumigatus, and Rhizopus arrhizus^41^. Overall, DyC-OCT can benefit disease diagnosis by enhancing cellular dynamic contrast to reveal changes in morphological features.

Another application of DyC-OCT is structure segmentation based on cell dynamics. For example, in the mouse tongue, DyC-OCT has successfully segmented the cornified layer, granular and spinous layers, basal layer, lamina propria, and muscle layer^26^. In retinal explants, the inner nuclear layer, outer nuclear layer, and photoreceptor layer have been clearly segmented by DyC-OCT^22,24,32^. In addition, DyC-OCT has improved accuracy in segmenting the urothelium in images of porcine urinary bladders^21^. For human heart organoids, our lab has reported the differentiation of cardiomyocytic beating clusters with two different beating frequencies using DyC-OCT^42^. The quick and reliable structure segmentation promotes the localization of clusters for quantitative assessment.

Another advantage of DyC-OCT is its ability to monitor morphological changes over time. For example, DyC-OCT has been used to monitor organoid development, such as retinal organoids^29^ and alveolar organoids^43^. DyC-OCT enables differentiation of cellular lifetime stages inside a single cell based on scattering particles’ movements in the organelles of HeLa cells^30^. Time-lapse DyC-OCT also shows the migration of immune cells inside airway tissues^16^. DyC-OCT provides a promising alternative to study the growth dynamics in organoids and tissue models, complementing existing microscopic imaging modalities.

### DyC-OCT as cell activity/viability assays

DyC-OCT has been applied to assess cell activity in response to different types of stimuli. In studies of age-related macular degeneration (AMD), DyC-OCT has monitored the wound healing process of primary porcine and human stem cell-derived retinal pigment epithelium (RPE) cells after scratch assays to study degenerative disease evolution^22^. In studying chemical stimulations, DyC-OCT has revealed an increased beating frequency of ciliated cells in airway tissues under ATP-γ-S stimulation, demonstrating the function of ATP-γ-S in strengthening metabolic activities^16^. DyC-OCT has captured a signal of dynamics decay in hepatocytes in a rat liver treated with a glycolysis inhibitor, indicating that the inhibitor suppressed the hepatocytes’ activity^15^. In alveolar organoids, DyC-OCT has revealed changes in the size and shape of alveoli after bleomycin was applied^43^. In plant science, DyC-OCT was applied to evaluate the effects of LED illumination as well as iron and phosphate deficiency on photosynthetic cells under various environmentally relevant stress challenges^28^.

Cell viability assays are important measurements to characterize cellular activity in response to external physical and chemical stimulations. Current methods rely on the introduction of luminescent probes that can be used to characterize cell membrane permeability, ATP synthetization, and protease activities, which are specific indicators of cellular viability. DyC-OCT measures cellular dynamics, which may be used as an alternative to measure cell viability. A recent study combined DyC-OCT with supervised machine learning models to quantitatively and in real-time assess the HeLa cell viability when keeping the cells at room temperature without CO_2_ supply for a few hours^44^.

DyC-OCT’s analytical potential extends to clinical applications, including drug screening^17,18^. Specifically, DyC-OCT was integrated into cancer research to assess the impacts of two anti-cancer drugs, Paclitaxel and Blebbistatin, on mammary organoids. This analysis revealed statistically significant distinctions between the time- and concentration-dependent reactions of the two drugs (Fig. 2c). In another study comparing Paclitaxel and the active metabolite of irinotecan (SN-38), DyC-OCT successfully captured the necrotic process of the cancer cells in human-derived tumor spheroids. These studies showed the promise of using DyC-OCT for drug screening.

## Outlook and conclusions

DyC-OCT has exhibited its potential to capture dynamic processes in various biological samples, the timescale of which contributes to the choice of hardware and imaging specifications. Molecular dynamics such as DNA replication and RNA transcription can happen in milliseconds^45^, while cellular differentiation and tissue development can take hours or longer^45^. It is important to choose an imaging protocol that matches the frame/volume rate of the OCT imaging system with the expected timescale/frequency of the dynamic activity. The frame/volume rate should be at least two times higher than the frequency of the activities to satisfy the Nyquist sampling criterion. Frame/volume rate can be changed by altering the exposure time or number of A-scans or B-scans in an image stack, while keeping into consideration the spatial sampling requirements. Cross-sectional OCT scanning speed restricts the frame rate to capturing events that occur on the millisecond scale, such as tissue metabolic process^46^. Slow activities happening in a fraction of second, such as beating of the heart organoid^47^ and ciliated cells in airway tissue or organoids, can be readily captured by DyC-OCT. For even slower processes that happen in minutes, hours or days, such as cell division, embryo development or tissue regeneration, time-lapse 3D DyC-OCT can be obtained using repeated 3D OCT scans.

Researchers have made impressive progress in DyC-OCT. However, a few challenges remain open. Box 1 summarized the key progress and opening challenges of DyC-OCT. Validation of observed imaging features is one of the main challenges in DyC-OCT research. DyC-OCT signals are not explicitly tied to any subcellular compartment (e.g., nucleus, cytoplasm) or specific molecules (e.g., DNA, fluorescence protein). They originate from temporal changes in activities which can vary between different cell and tissue types. As a result, without co-registered images from other biological imaging technologies as validation, interpretation of DyC-OCT signals can be challenging. Although both histology^25,48^ and fluorescence microscopy^18,20,48^ have been applied for comparison with DyC-OCT results, co-registration between the modalities remains a challenge, typically due to different imaging orientation between 3D OCT volumes and 2D imaging modalities^48,49^. The difficulties in co-registering also bring obstacles in precise validation based on quantitative matching, which requires pixel-wise registration between images from DyC-OCT and other modalities. The lack of validation prevents DyC-OCT from being applied to quantitatively analyze tissue morphology and cellular activities. Multi-modal imaging platforms that combine DyC-OCT with modalities like confocal, light-sheet, or two-photon microscopy enable co-registered imaging of the same sample. This approach not only cross-validates DyC-OCT results but also yields complementary information. For example, simultaneous calcium fluorescence and DyC-OCT imaging of beating heart organoids can enhance our understanding of cardiac electromechanical coupling^42^. Moreover, correlating DyC-OCT signals with established biological activities aids in interpreting DyC-OCT data and linking it to specific dynamic events. Researchers have observed OCT fluctuation pattern changes following stimulations known to alter activities, like ATP-γ-S^16^, inhibitors^15,43^ and cancer drugs^18,48^. These findings reinforce DyC-OCT’s validity and provide insights into dynamic activities in biological systems.

Another challenge is that despite fast OCT B-scan acquisition, DyC-OCT imaging, especially 3D DyC-OCT, requires dozens or hundreds of repeated frames scanning from all B-scan locations, prolonging the imaging acquisition time. To speed up DyC-OCT acquisition, one potential solution is to use parallel OCT imaging, such as space-division multiplexing OCT (SDM-OCT)^50^ or line-field parallel SS-OCT^51^, which acquire OCT images from different B-scan locations simultaneously. Reducing acquisition time not only enhances the sampling rate of 3D DyC-OCT but also opens up possibilities for in vivo applications, where fast acquisition is essential to minimize motion artifacts.

Although classical algorithms, such as LIV, STD and FFT, can extract the dynamic pattern from OCT signal fluctuations, their image processing steps are laborious. Alternatively, machine-learning or deep-learning-based models can extract dynamic features to generate DyC-OCT images and reveal dynamic activities not resolvable by classical algorithms. Learning-based time series analysis algorithms, such as long short-term memory (LSTM)^52^ networks, could be employed to extract more informative dynamic patterns.

In summary, DyC-OCT leverages high-resolution OCT imaging to capture unique cellular dynamics and activities in live samples, without requiring tissue labeling. Various algorithms have been developed to identify distinct motion patterns, thus providing dynamic insights into biological tissues. DyC-OCT’s application extends to dynamic image contrast enhancement in ex vivo tissue samples, 3D organoids, and other in vitro models. Future advancements in multi-modal imaging systems, high-speed and parallel OCT imaging, combined with cutting-edge image processing algorithms, promise to fully unveil the capabilities of this emerging imaging technology.

Box 1 Key progress and open challenges Key Progress

1. DyC-OCT provides noninvasive, high-resolution, contrast-enhanced imaging by utilizing diverse pattern extraction algorithms across common OCT modalities.
2. DyC-OCT discerns cellular microstructures in ex vivo tissues and in vitro organoids. This technique has been utilized in disease diagnosis, structure segmentation, and developmental monitoring.
3. DyC-OCT provides quantitative information on dynamic biological activities and has been employed as potential cellular activity/viability assays.

Open Challenges and Opportunities

1. Interpreting features in DyC-OCT images is challenging without rigorous validation through established methods, owing to the indirect relationship between DyC-OCT signals and the dynamic biological activities of cells and tissues.
2. To date, DyC-OCT applications have primarily focused on ex vivo living tissues. In vivo applications face challenges due to prolonged image acquisition times and sample motions. These issues could potentially be addressed with high-speed parallel imaging OCT systems.
3. Learning-based algorithms may be able to effectively extract dynamic features for DyC-OCT, thereby improving efficiency and revealing dynamic activities that classical algorithms cannot resolve.

### Supplementary information

Supplementary Table 1
