# Supplementary material for: Dynamic contrast optical coherence tomography (DyC-OCT) for label-free live cell imaging
Source: Commun Biol. 2024 Mar 6;7:278. doi: 10.1038/s42003-024-05973-5 (PMC10918170; doi:10.1038/s42003-024-05973-5)
Supplement: Supplementary file 1 — Supplementary Table 1 [file 42003_2024_5973_MOESM1_ESM.pdf]

Supplementary Table 1: Summary of DyC-OCT processing algorithms on time complexity, pattern extracted, number of repeated frames required, advantages and drawback.

Algorithms	Time Complexity (N repeated)	Extracted Parameters	Repeated frames	Advantages	Drawbacks
**STD/LIV**	O(N)	Amplitude fluctuations	~5-10	Rapid image acquisition, low memory costs and short processing time	Amplitude information only
**OCDS**	O(N)	De-correlation time	~10-20		Signal changing speed information only
**PSD**	O(NlogN)	Frequency & amplitude	>100	Multiple patterns extraction to differentiate more types of structures	High acquisition and processing time cost
